# Therapeutic effects of STAT3 decoy oligodeoxynucleotide on human lung cancer in xenograft mice

**DOI:** 10.1186/1471-2407-7-149

**Published:** 2007-08-04

**Authors:** Xulong Zhang, Jian Zhang, Lihua Wang, Haiming Wei, Zhigang Tian

**Affiliations:** 1 Institute of Immunopharmacology & Immunotherapy, School of Pharmaceutical Sciences, Shandong University, Jinan 250012, China; 2 Institute of Immunology, School of Life Sciences, University of Science and Technology of China, Hefei 230027, China

## Abstract

**Background:**

Signal transducer and activator of transcription 3 (STAT3) is usually constitutively activated in a variety of malignancies. Therefore, STAT3 may be a promising target for treatment of tumor cells. To explore the possibility of a double-stranded decoy oligodeoxynucleotide (ODN) targeted blocking STAT3 over-activated tumor cells, we, here, evaluate the efficacy of STAT3 decoy ODN on human lung cancer cells *in vitro *and *in vivo*.

**Methods:**

A STAT3 decoy ODN was transfected into A549 lung cancer cell line *in vitro *by using lipofectamine. The flow cytometry and fluorescent microscopy were used to detect the transfection efficiency and the sub-cellular localization of STAT3 decoy ODN in A549 cells. Cell proliferation was determined by counting cell numbers and [^3^H]-thymidine uptake. Cell apoptosis was examined with Annexin V and propidum iodide by flow cytometry. The expression levels of STAT3 target genes were identified by RT-PCR and immunoblot. For *in vivo *experiment, A549 lung carcinoma-nude mice xenograft was used as a model to examine the effect of the STAT3 decoy by intratumoral injection. At the end of treatment, TUNEL and immunohistochemistry were used to examine the apoptosis and the expression levels of bcl-xl and cyclin D1 in tumor tissues.

**Results:**

STAT3 decoy ODN was effectively transfected into A549 lung cancer cells and mainly located in nucleus. STAT3-decoy ODN significantly induced apoptosis and reduced [^3^H]-thymidine incorporation of A549 cells as well as down-regulated STAT3-target genes *in vitro*. STAT3 decoy ODN also dramatically inhibited the lung tumor growth in xenografted nude mice and decreased gene expression of bcl-xl and cyclin D1.

**Conclusion:**

STAT3 decoy ODN significantly suppressed lung cancer cells *in vitro *and *in vivo*, indicating that STAT3 decoy ODN may be a potential therapeutic approach for treatment of lung cancer.

## Background

The signal transducers and activators of transcription 3 (STAT3), a member of a transcription factor family of seven proteins (STAT1, 2, 3, 4, 5a, 5b, and 6), plays important roles in regulating cell growth, differentiation, apoptosis, angiogenesis, and immune responses [1,2]. STAT3 transduces extracellular signals made by numerous cytokines and growth factors to nucleus and directly regulate gene transcription [3]. Cytokines (e.g. interleukin-6) and growth factors (e.g. epidermal growth factor) may bind to their cognate receptors and then activate tyrosine kinases, which phosphorylates the tyrosine residue of STAT3. Upon activation, STAT3 dimers translocate into the nucleus, where they bind to STAT3 specific DNA response elements and activate their transcription [4,5].

Dysregulation and constitutive activation of STAT3 have been found in numerous primary cancers such as lymphomas, leukemias, multiple myelomas, prostate, breast, lung, head and neck, melanoma, pancreas, ovary and gastric cancer cells [6-16]. Lung cancer is the leading cause of death in cancer in the United States. In 2006, an estimated 174,470 new cases, accounting for about 12% of cancer diagnoses and an estimated 162,460 deaths, accounting for about 29% of all cancer deaths, are expected to occur (The American Cancer Society). Constitutive activation of STAT3 correlates with cell proliferation in non-small-cell lung cancer (NSCLC) and also inhibits apoptosis [11,17]. Restoration of suppressors of cytokine signalling-3 (SOCS-3), which is frequently silenced by hypermethylation in lung cancer cells, resulted in the down-regulation of activated STAT3, leading to induction of apoptosis and growth suppression [18].

The constantly activated STAT3 contributes to oncogenesis by up-regulation of genes encoding bcl-xl, bcl-2, c-myc, cyclin D1, survivin, mcl-1 [7,8,16,19], and VEGF, IL-10, TGF-β et al [20-22], which can protect apoptosis, enhance cell proliferation, promote angiogenesis and evade immune surveillance [1]. Though there are multiple oncogenic signaling pathways in each individual tumor, blockade of STAT3 signaling is often sufficient to induce growth arrest and apoptosis in many different tumors [8-12,16]. Therefore, the association of STAT3 activation with tumor progression suggests that STAT3 may be an attractive molecular target for cancer therapy.

Many methods to block STAT3 activation were developed including RNA antisense, RNA interference (RNAi) and dominant negative mutants [9,23,24]. A double-stranded decoy oligodeoxynucleotide (dsODN) against transcription factor [25] is based on the competition between the endogenous *cis*-elements within the regulatory regions of target genes and the exogenously added molecules mimicking the specific *cis*-elements [26]. Transfection of dsODN will result in the attenuation of the authentic interactions of STAT3 with their cis-elements and subsequent alteration of gene expression. This strategy has been successfully used for inhibition of STAT3 in head and neck cancer and also for inactivation of STAT6 in shifting IL-4-driven Th2 cell activity [27,28]. Recently, we have found that STAT3 decoy ODN could inhibit cell growth of a pulmonary giant cell carcinoma cell line PG *in vitro *[29].

In the present study, we demonstrate that STAT3 decoy ODN can suppress lung cancer cells *in vitro *and *in vivo *by efficiently blocking STAT3 signaling, suggesting the therapeutic potential of STAT3 decoy ODN in treatment of human lung carcinoma.

## Methods

### Cell line and cell culture

The human non-small-cell-lung cancer line, A549, were grown in RPMI 1640 medium (GIBCO/BRL) supplemented with 10% fetal bovine serum (FBS), 100 IU/ml penicillin and 100 μg/ml streptomycin in 5% CO_2 _at 37°C.

### Antibodies and reagents

Anti-STAT3, anti-phospho-specific STAT3 (Tyr705, Ser727), anti-bcl-xl, anti-cyclinD1, anti-β-actin antibodies and horseradish peroxidase-conjugated second antibody were purchased from Cell Signaling Technology (New England BioLabs Inc.). Annexin V-FITC was obtained from BD Biosciences (BD PharMingen, San Diego, CA).

### STAT3 decoy and scramble ODN

Phosphorothioated sense and antisense strands of STAT3 decoy or scramble ODNs were synthesized by Expedite™ Nucletic Acid Synthesis System (TaKaRa Biotechnology, Dalian). The STAT3 decoy ODN sequence was 5'-CATTTCCCGTAAATC-3', 3'-GTAAAGGGCATTTAG-5' and the scramble ODN sequence was 5'-CATCTTGCCAATATC-3', 3'-GTAGAACGGTTATAG-5' [27,30]. The sense and antisense strands were annealed and purified by HPLC.

### Transfection of ODN

Transfections were carried out by lipofectamine 2000 (Invitrogen life technologies, Carlsbad, CA) according to the manufacturer's instructions. Briefly, cells were seeded at a density of 7 × 10^3 ^cells/well (400 μl) in 48 well plates (Costar, Corning, NY) for 24 h. After washing once with PBS, cells were transfected with lipofectamine 2000/control, lipofectamine 2000/decoy ODN, or lipofectamine 2000/scramble ODN complexes. The final concentration of ODN was 50, 25 and 12.5 nmol/L separately.

### Flow cytometry and fluorescent microscope

The decoy and scramble ODN were labeled with FITC at 5' of ODNs (TaKaRa Biotechnology, Dalian). After transfection of FITC-ODN, cells were detached, washed with PBS extensively and detected by flow cytometry (FACSCalibur, BD Biosciences, CA). For ODN location analysis, transfected cells were washed three times, fixed and permeabilized, nuclei were stained with DAPI (Jingmei Biotechnologies, China) for 10 min at room temperature. After washing, the cells were observed under a fluorescent microscope.

### Cell proliferation assay

Cells were plated and transfected with decoy or scramble ODN as described above. Cell number was determined by counting with a hemocytometer using trypan blue exclusion. Above 90% viability was regarded as no toxicity. Cell proliferation was examined by measuring DNA synthesis using tritiated thymidine (^3^H-dThd) uptake. Before transfection, cells (4 × 10^4^/well) were cultured in a 24-well plate in growth media for 24 h. STAT3 decoy or scramble ODN were transfected for 18 h separately, and then pulsed for the remaining 6 h with [^3^H]-thymidine (3.75 μCi/ml). [^3^H]-thymidine incorporation was analyzed by liquid scintillation counting.

### Cell apoptosis assay

Twelve hours after transfection of 25 nmol/L STAT3 decoy or scramble ODN, cells were detached, washed and resuspended in 100 μl Annexin V binding buffer, and subsequently stained with Annexin V-FITC and propidum iodide (Sigma) according to the manufacturer's recommendation. Data acquisition and analysis were performed by a flow cytometry using CellQuest software.

### Isolation of Total RNA and reverse transcription (RT)-polymerase chain reaction (PCR)

RT-PCR was performed as previously described [31]. Briefly, total RNA was isolated using TRIzol (Invitrogen, Carlsbad, CA) and subjected for reverse transcription using M-MLV Reverse Transcriptase (Invitrogen, Carlsbad, CA) according to the manufacturer's instructions. cDNAs were then amplified with the corresponding gene-specific primers which synthesized by Shanghai Genecore Biotechnologies (Shanghai, China)(table 1). The PCR products were electrophoresed and photographed using AlphaEaseFC. The bands were examined by densitometry using AlphaEaseFC software with normalization of each band to their corresponding loading control.

**Table 1:** RT-PCR primers for STAT3 target genes

**genes**	**Primer sequences**	**Ta**	**Product size**
β-actin	5'-CTC CTT AAT GTC ACG CAC GAT TT-3'	56°C	539 bp
	5'-GTG GGG CGC CCC AGG CAC CA-3'		
cyclin D1	5'-GAG ACC ATC CCC CTG ACG GC-3'	58°C	484 bp
	5'-CTC TTC CTC CTC CTC GGC GGC-3'		
bcl-xl	5'-GGA AAG CGT AGA CAA GGA GAT GC-3'	55°C	236 bp
	5'-GGT GGG AGG GTA GAG TGG ATG GT-3'		
c-myc	5'-GGT CTT CCC CTA CCC TCT CAA CGA-3'	55°C	386 bp
	5'-GGC AGC AGG ATA GTC CTT CCG AGT-3'		
mcl-1	5'-GTG GTG GTG GTG GTT GGT TA-3'	54.5°C	575 bp
	5'-CGG CAG TCG CTG GAG ATT AT-3'		
survivin	5'-GCA TGG GTG CCC CGA CGT TG-3'	55°C	446 bp
	5'-GCT CCG GCC AGA GGC CTC AA-3'		
cyclinE	5'-ATA CAG ACC CAC AGA GAC AG-3'	54.5°C	301 bp
	5'-TGC CAT CCA CAG AAA TAC TT-3'		

Reprinted with permission from Oncology Reports [29].

### Western blot

Sodium dodecyl sulfate polyacrylamide gel electrophoresis (SDS-PAGE) and immunoblotting were processed as described previously [31]. Briefly, the whole cells were lysed in lysing buffer after transfected with STAT3 decoy ODN for 24 h. The whole cell extracts (30 μg/lane) were separated by SDS-PAGE and transferred to nitrocellulose membrane. After blocked in Tris-buffered saline with 5% (w/v) nonfat dry milk, membranes were incubated with primary antibodies (1:1000 dilution) according to the manufacturer's instructions and then incubated with horseradish peroxidase conjugated secondary antibody. The proteins were detected by the enhanced chemiluminescence (ECL) system (Pierce, Rockford, IL) using X-ray film. The bands were examined by densitometry using AlphaEaseFC software (Version 4.0.0, Alpha Innotech Corporation) with normalization of each band to their corresponding loading control.

### Human tumor-bearing nude mouse

Female athymic nude mice (Balb-nu/nu, 4 weeks old, weighing 18 ± 2 g) were purchased from the Shanghai Experimental Animal Center (Chinese Academy of Sciences, Shanghai), and maintained at an animal facility under pathogen-free conditions. The handling of mice and experimental procedures were conducted in accordance with experimental animal guidelines. A549 cells were harvested and re-suspended in RPMI 1640 media. 8 × 10^6 ^cells (150 μl) were injected into the right flank of the mice subcutaneously. About 7 days later, when the tumors reached about 35 mm^3^, the mice were randomly assigned to three groups with six mice in each group (PBS treatment control, STAT3 scramble ODN treatment control and STAT3 decoy ODN treatment group). ODN (25 μg in 25 μl PBS) was intratumorally injected every day for totally 30 days. During this period, the tumor volume was determined by measuring the length (l) and the width (w) every 5 days, and calculated by using the function (V = l w^2^/2).

### Terminal Deoxynucleotidyl Transferase-mediated Nick End Labeling (TUNEL)

A549 xenografts were collected on the termination day of antitumor experiment, sectioned and stained for apoptotic cells by TUNEL using the In Situ Cell Death Detection Kit, POD (Roche Applied Science, Germany) according to manufacturer's guides. Briefly, frozen sections were fixed in 4% paraformaldehyde, incubated in blocking solution and in permeabilisation solution, and incubated in TUNEL reaction mixture for 60 min at 37°C. After rinsing, the sections were incubated with Converter-POD for 30 min at 37°C and developed with DAB Substate Kit (Boster, Wuhan). The slides were lightly counterstained with hematoxylin and then dehydrated and mounted. For each group, 4 samples were evaluated and 5–10 fields of view were quantitated on each section. The number of apoptotic cells per high power field (HPF) was counted.

### Immunohistochemistry

The immunohistochemical staining was performed using peroxidase labeled streptavidin-biotin method (Histostain plus kits, ZYMED, CA) according to the manufacturer's protocol. Briefly, five-μm frozen sections were fixed in acetone. Endogenous peroxidase was blocked by 3% hydrogen peroxide. After blocked with normal goat serum, diluted bcl-xl and cyclin D1 primary antibodies (at a dilution of 1:300) were added to completely cover tissue and incubated in humidified chamber overnight at 4°C, and then incubated with biotinylated second antibody, and finally incubated with streptavidin-peroxidase conjugate (S-A/HRP). The immune complex was visualized with 3, 3-diaminobenzidine (DAB substate kit, Boster, Wuhan). The slides were lightly counterstained with hematoxylin and then dehydrated and mounted. As negative controls, rabbit immunoglobulin was used to replace primary antibody.

### Statistical analysis

Statistical analysis was performed with SPSS software (version 10.0, SPSS Inc). Data were presented as mean ± SD. *p *< 0.05 were considered statistically significant.

## Results

### STAT3 was constitutively activated in A549 human lung cancer cell line and STAT3 decoy ODN was efficiently transfected into A549 cell lines

**Figure 1:**
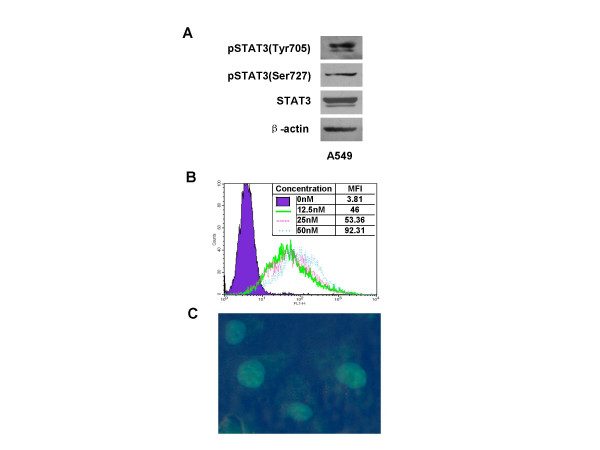
**STAT3 was constitutively activated in A549 human lung cancer cell line and the efficiency of STAT3 decoy ODN transfected into A549 cells**. **(A) **The A549 lung cancer cells were lysed in lysing buffer and the whole cell extracts (30 μg/lane) were separated by SDS-PAGE and then examined by western blotting using anti-STAT3, anti-phospho-specific STAT3 (Tyr 705, Ser727) or β-actin antibodies. The proteins were then detected by the enhanced chemiluminescence (ECL) system by exposing to X-ray film. (**B) **A549 cells were transfected with FITC-labeled STAT3 decoy ODN at final concentration of 12.5, 25 and 50 nmol/L in presence of lipofectamine 2000. After transfected for 6 h, the cells were examined by flow cytometry. **(C) **After transfected with 25 nmol/L FITC-labeled STAT3-ODN for 6 h, the cells were fixed, permeabilizated and stained with DAPI, and then fluorescent microscopy was used to observe the nucleus location of ODN. STAT3 decoy ODN (green) and nuclei (blue) were overlaid with their contrast image.

To examine whether STAT3 is persistently activated in human lung cancer cells, the whole cell lysate from the human non-small-cell-lung carcinoma line A549 was processed for western blotting with anit-STAT3 and anti-phospho-specific STAT3 (Tyr705, Ser727) antibodies. As shown in Fig. 1A, STAT3 was phosphorylated on Tyr705 and Ser727 in A549 lung cancer cells, which was consistent with our previous observation in human pulmonary giant cell carcinoma cell line PG [29]. These data demonstrated that STAT3 was constitutively activated in human lung cancer cells.

To assay the transfection efficiency of decoy ODN into cells, we used a FITC-labeled decoy ODN to verify whether the decoy ODN could be introduced into A549 cells. The results measured by a flow cytometry showed the decoy ODN could be efficiently transfected into A549 cells with a dose-dependent manner after incubating for 6 h (Fig. 1B). The transfection efficiency was 93%, and the mean fluorescence intensity (MFI) was 53.36 at 25 nmol/L, and 92.31 at 50 nmol/L of ODN, respectively. Similar results were observed with scramble ODN. Moreover, the sub-cellular localization of decoy ODN was determined by a fluorescent microscopy. As shown in Fig. 1C, most of decoy ODN were located in nucleus. These data indicated that decoy ODN could be efficiently transfected into cells and located in the nucleus.

### STAT3 decoy ODN inhibited the growth of A549 cells *in vitro*

**Figure 2:**
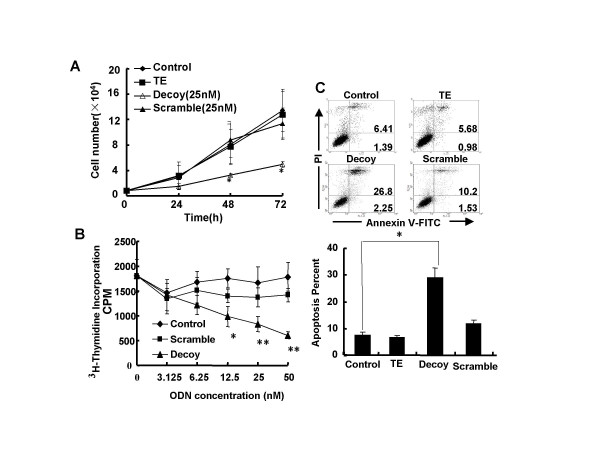
**Inhibitory effect of STAT3 decoy ODN on A549 lung cancer cells *in vitro***. **(A) **A549 cells were seeded at a density of 1 × 10^4 ^cells per well in 48 well plates. After 24 h, the cells were treated with 25 nmol/L STAT3 decoy ODN, STAT3 scramble ODN or vehicle control (TE) for different time using lipofectamine 2000. The cumulative number of cells was accounted using trypan blue exclusion. Values are expressed as mean ± SD of three independent experiments. **(B) **A549 cells (4 × 10^4^/well) were treated with STAT3 decoy ODN or scramble decoy ODN for 18 hours at 37°C, and then pulsed with [^3^H]-thymidine for an additional 6 hours. The incorporation of [^3^H]-thymidine was analyzed by liquid scintillation counting. **(C) **A549 cells were plated in six well plate, after transfected with 25 nmol/L STAT3 decoy ODN or scramble ODN, the apoptotic A549 cells were detected by flow cytometry using Annexin V-FITC and PI. The histogram represented the percentage of apoptotic cells. Values are expressed as the mean ± SD of three independent experiments. Statistical significance was determined as *p < 0.01 compared to other groups.

Since cancer is now viewed not only as being the consequence of uncontrolled proliferation, but also as the result of an altered balance between cell proliferation and rate of apoptosis, we examined the effects of STAT3 decoy ODN on cell proliferation and apoptosis of lung cancer cells. A549 cells were transfected with STAT3 decoy ODN or scramble control ODN. As present in Fig. 2A, the total amount of A549 cells was significantly reduced by STAT3 decoy ODN (25 nmol/L) with maximum inhibition rate of 63.45% (*p *< 0.01). STAT3 decoy ODN also markedly suppressed the incorporation of [^3^H]-thymidine (65.9%) if compared with the control cells (Fig. 2B). In contrast, the scramble did not inhibit the growth of human lung cancer cells. Cell apoptosis was also examined with Annexin V and PI by flow cytometry after cells transfected with STAT3 decoy ODN. Compared with scramble ODN, STAT3 decoy ODN could significantly induce apoptosis from 11.73% to 29.05% (Fig. 2C, *p *< 0.01). These findings suggest the STAT3 decoy effectively inhibits the cell growth by reducing proliferation or/and inducing apoptosis of lung carcinoma cells.

### STAT3 decoy ODN inhibited tumor growth and increased apoptosis in xenografts

**Figure 3:**
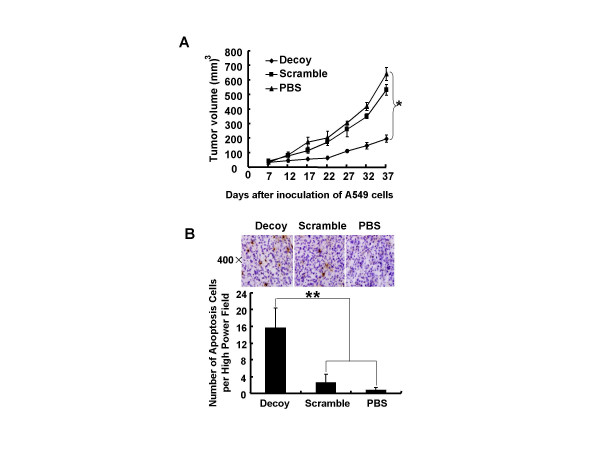
**Inhibitory effect of STAT3-decoy-ODN on A549 tumor – bearing nude mice**. **(A) **Human A549 tumor-bearing nude mice were established as described in materials and methods. The mice were intratumorally injected daily with STAT3 decoy ODN (25 μg), scramble ODN (25 μg) or vehicle control (25 μl PBS) for total thirty injections, and the tumor volumes were measured at the indicated day. *p < 0.01 versus PBS treatment control. **(B) **After daily intratumoral injection of 25 μg STAT3 decoy ODN for thirty times, the xenografts were collected, sectioned and stained for apoptotic cells by TUNEL which performed as described in materials and methods. The brown stain represented the DNA fragmentation of apoptotic cells and the blue showed the nuclei stain with hematoxylin (original magnification ×400). The cumulative results showed the average number of apoptotic cells per High Power Field. **p < 0.001 versus PBS treatment control.

The *in vivo *efficacy of STAT3 decoy ODN was tested using nude mice bearing human A549 lung carcinoma xenografts. When the tumors were established, the mice were randomly assigned and treated with STAT3 decoy or scramble ODN (intratumoral injection daily). The tumor growth curves were shown in Fig. 3A. After approximately 30 days, the tumor volume in PBS control group increased to 639 ± 43 mm^3^, whereas the tumor volume in STAT3 decoy ODN treatment group was only 195 ± 24 mm^3^, indicating that the tumor growth was inhibited by 69.5% (*p *< 0.01). TUNEL was also used to examine the apoptotic tumor cells after STAT3 decoy ODN treatment *in vivo*. As shown in Fig. 3B, the number of TUNEL positive cells in tumor tissues treated with STAT3 decoy ODN was significantly greater than that treated with vehicle or scramble ODN. Compared with PBS treatment group, the apoptotic cells in STAT3 decoy ODN treatment group were increased by about 20 folds (*p *< 0.001). These results indicate that STAT3 decoy ODN is effective in reducing tumor growth and inducing apoptosis of lung tumor cells in mice.

### STAT3 decoy ODN decreased the expression of STAT3-regulated cell growth or anti-apoptotic genes of lung cancer cells in culture

**Figure 4:**
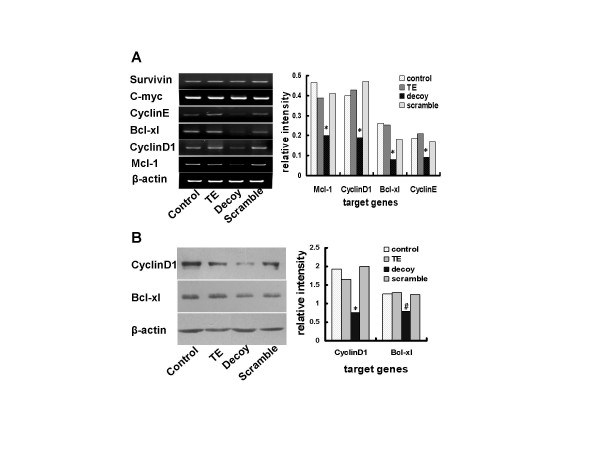
**STAT3 decoy ODN decreased the expression of STAT3-regulated cell growth or anti-apoptotic genes**. **(A) **After transfected with 25 nmol/L STAT3 decoy ODN or scramble ODN for 24 h, the whole A549 cells were harvested and the total cellular RNA was isolated using Trizol Reagent, mRNA levels of STAT3-correlated genes such as mcl-1, cyclin D1, bcl-xl, cyclinE, c-myc and survivin were detected using RT-PCR method. The PCR products were electrophoresed and photographed using AlphaEaseFC. **(B) **After transfected with 25 nmol/L decoy ODN, the whole A549 were harvested and the whole-cell extracts were obtained. And western blotting for bcl-x1 and cyclin D1 were then examined as described in materials and methods. Histogram presented the relative expression level of each gene or protein after normalization to its corresponding internal control. Statistical significance was determined as *p < 0.01 and ^#^*p *< 0.05 compared to control.

To investigate whether the inhibitory effect of STAT3 decoy ODN on lung carcinoma cells is correlated with expression of STAT3-target genes, RT-PCR was used to detect the mRNA levels of STAT3 target genes such as cell cycle genes cyclin D1 and cyclin E, or apoptosis regulating genes mcl-1 and bcl-xl. As illustrated in Fig. 4A, the mRNA levels of mcl-1, cyclin D1, bcl-xl and cyclin E were decreased by 57.1%, 52.3%, 69.5% and 50%, respectively (*p *< 0.01), however, the transcription of c-myc and survivin did not show obvious changes. By contrast, scramble ODN showed no effect on the transcription of the cancer cells. Since cyclin D1 and bcl-xl are thought to be the downstream proteins of activated STAT3 [19], which greatly contributes to tumor progression, we then examined the expression levels of these two proteins by western blot. Compared with control, the expression levels of bcl-xl and cyclin D1 in A549 cells were attenuated by 37.4% (*p *< 0.05) and 60.7% (*p *< 0.01), respectively (Fig. 4B). These data suggest that blockade of the expression of STAT3-drived cell growth and apoptosis-related genes may be involved in the inhibition of STAT3 decoy ODN on growth of human lung cancer cells in culture.

### STAT3 decoy ODN decreased the expression of bcl-xl and cyclin D1 in xenografts by Immunohistochemical analysis

**Figure 5:**
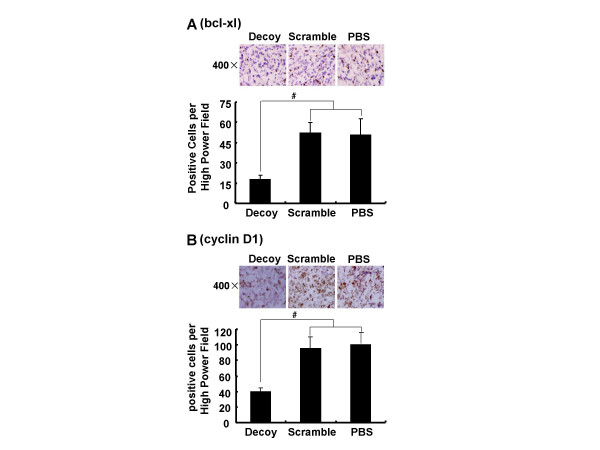
**STAT3 decoy ODN decreased the expression of bcl-xl and cyclin D1 in xenografts by Immunohistochemical analysis**. The harvested xenografts which treated with decoy or scramble ODN were sectioned and performed immunohistochemical analysis of bcl-xl (**A**) and cyclin D1 (**B**) as described in materials and methods. The brown showed the expression levels of bcl-xl and cycln D1; the blue showed the nuclei (original magnification ×400). Each histogram presented the number of bcl-xl and cyclin D1 positive cells per High Power Field in tumor tissues. Statistical significance was determined as ^#^*p *< 0.05.

To evaluate effect of STAT3 decoy ODN on gene expression in nude mice with A549-derived xenografts, immunohistochemical analysis was practiced to determine the expression of bcl-xl and cyclin D1 in tumor tissues. The intensity of brown stain corresponds to the expression levels of bcl-xl (Fig. 5A) and cyclin D1 (Fig. 5B). The level of bcl-xl and cyclin D1 was lower in STAT3 decoy ODN-treated tumor-bearing mice compared with that in vehicle control or scramble control (*p *< 0.05). These observations on tumor tissues from *in vivo *xenografts were consistent with the data obtained from in culture experiments (Fig. 4A &4B).

## Discussion

Cumulative evidence support that STAT3 activation and the novel decoy ODN approach may be used as an antitumor strategy. Unlike in normal cells and tissues, the constitutively activated STAT3 has been observed in a wide variety of human cancer cell lines and primary tumors. Therefore, STAT3 has also been recognized as an oncogene [2,19]. By using the specific antibody against phosphorylated STAT3 and western blot, we, here, show that STAT3 is constitutively activated in A549 human non-small-cell lung cancer cell line (Fig. 1A), which was consistent with our previous observations [29] in human pulmonary giant cell carcinoma PG cell line. These results further support the idea that the activated STAT3 may be a good molecular target for lung cancer treatment.

Decoy ODNs have been proposed as a useful approach to block the function of transcription factors. There are several attractive advantages of decoy method over the other gene therapeutic approaches [32,33]. First, decoy ODN, as a small DNA molecule, can be easily delivered to specific tissues and transfected into cells, and directly abrogate the activated transcript factors. Second, a growing number of transcript factors with their promoter sequences have been found, which made the potential drug targets plentiful and readily identifiable. Third, the synthesis, storage and transportation of decoy ODNs are much simpler than other approaches. Finally, decoy ODNs have been documented to be more effective than antisense ODN in blocking constitutively activated transcript factors [26]. Moreover, the clinical application of decoy ODN against E2F was approved by the Food and Drug Administration (FDA) to treat neointimal hyperplasia in vein bypass grafts. A clinical trial using NF-κB decoy ODN was also started in Japan, and positive results of phase I/II trial for the treatment of atopic dermatitis was made in 2006.

In present study, we used STAT3 decoy ODN to turn down the constitutively activated STAT3 in lung cancer cells. The results showed that STAT3 decoy ODN significantly decreased viable cell number by inducing apoptosis and reducing [^3^H]-thymidine incorporation of A549 non-small-cell lung cancer cell line *in vitro *(Fig. 2), which was in parallel with our recent findings in a different type of lung cancer cell line PG [29], suggesting that STAT3 decoy could effectively suppress cell growth of lung cancer. Since either cells that are dead or undergoing apoptosis, like cells that are stuck in G0/G1, may also show reduced [^3^H]-thymidine incorporation compared with control cells, further studies are necessary to clarify what the relative contributions of apoptosis *vs*. reduced proliferation are to the growth inhibition observed in the lung cancer cells.

To confirm the above inhibitory effect of STAT3 decoy ODN on human lung cancer cells in culture, we used human A549 lung tumor xenograft mice to assay the in vivo efficacy of STAT3 decoy ODN. Compared to PBS vehicle control and scramble control, STAT3 decoy ODN significantly inhibited the tumor growth (Fig. 3A) in mice as well as remarkably increased the number of TUNEL-positive apoptotic cells in xenograft tumor tissues (Fig. 3B). Both *in vitro *and *in vivo *experimental results demonstrated the potential values of STAT3 decoy ODN approach in the treatment of lung cancer.

We next investigated the molecular mechanisms by which STAT3 decoy ODN inhibited growth of lung cancer cells. Blockade of STAT3 signaling by STAT3 decoy ODN could down-regulate the cell growth genes (such as cyclin D1) or anti-apoptotic genes (such as bcl-xl) at both mRNA and protein levels (Fig.4 &5). Inhibition of STAT3 signalling resulted in the decreased survivin and c-myc expression in many tumor cell lines, however, in A549 cells, the transcription levels of survivin and c-myc were not down-regulated by STAT3 decoy ODN treatment. One of the reasons was that the expressions of c-myc and survivin may also be controlled by other transcription factors such as STAT5 and STAT1, which may compensate it when the STAT3 was blocked [34,35]. Therefore, the inhibition of tumor growth by STAT3 decoy ODN was significantly associated with blockade of the STAT3-regulated cell cycle and anti-apoptotic proteins.

## Conclusion

In summary, we have provided evidence that STAT3 decoy ODN suppresses growth of lung cancer cells *in vitro *and *in vivo *through affecting the balance between cell proliferation and rate of apoptosis. This inhibitory effect may be correlated to blockade of STAT3-regulated cell cycle genes or anti-apoptotic genes. Our findings highlight that STAT3 may be a good candidate molecular target, and STAT3 decoy ODN may potentially be used as an anti-lung cancer therapeutic small bio-molecule.

## Abbreviations

STAT: signal transducer and activator of transcription; ODN: oligodeoxynucleotide; ds: double-stranded; TFD: transcription factor decoy; VEGF: vascular endothelial growth factor; IL: interleukin; TGF-β: transforming growth factor-β.

## Competing interests

The author(s) declare that they have no competing interests.

## Authors' contributions

XLZ, JZ and HMW performed almost all the experiments. XLZ analyzed the data and drafted the manuscript. LHW advised *in vitro *experiments. HMW advised and assisted in vivo experiments. JZ and ZGT conceived and supervised the study and revised the manuscript. All authors read and approved the final manuscript.

## Pre-publication history

The pre-publication history for this paper can be accessed here:


